# The Solvent Effect on Composition and Dimensionality of Mercury(II) Complexes with Picolinic Acid

**DOI:** 10.3390/molecules26165002

**Published:** 2021-08-18

**Authors:** Željka Soldin, Boris-Marko Kukovec, Dubravka Matković-Čalogović, Zora Popović

**Affiliations:** 1 Division of General and Inorganic Chemistry, Department of Chemistry, Faculty of Science, University of Zagreb, Horvatovac 102a, HR-10000 Zagreb, Croatia; dubravka@chem.pmf.hr (D.M.-Č.); zpopovic@chem.pmf.hr (Z.P.); 2 Department of Physical Chemistry, Faculty of Chemistry and Technology, University of Split, Ruđera Boškovića 35, HR-21000 Split, Croatia

**Keywords:** mercury(II), picolinic acid, coordination polymer, mononuclear complex, solvent type, metal-to-ligand ratio

## Abstract

Three new mercury(II) coordination compounds, {[HgCl(pic)]}*_n_* (**1**), [HgCl(pic)(picH)] (**2**), and [HgBr(pic)(picH)] (**3**) (picH = pyridine-2-carboxylic acid, picolinic acid) were prepared by reactions of the corresponding mercury(II) halides and picolinic acid in an aqueous (**1**) or alcohol–methanol or ethanol (**2** and **3**) solutions. Two different types of coordination compounds were obtained depending on the solvent used. The crystal structures were determined by the single-crystal X-ray structural analysis. Compound **1** is a one-dimensional (1-D) coordination polymer with mercury(II) ions bridged by chelating and bridging *N*,*O*,*O*′-picolinate ions. Each mercury(II) ion is four-coordinated with a bidentate picolinate ion, a carboxylate O atom from the symmetry-related picolinate ion and with a chloride ion; the resulting coordination environment can be described as a highly distorted tetrahedron. Compounds **2** and **3** are isostructural mononuclear coordination compounds, each mercury(II) ion being coordinated with the respective halide ion, *N*,*O*-bidentate picolinate ion, and *N*,*O*-bidentate picolinic acid in a highly distorted square-pyramidal coordination environment. Compounds **1–3** were characterized by IR spectroscopy, PXRD, and thermal methods (TGA/DSC) in the solid state and by ^1^H and ^13^C NMR spectroscopy in the DMSO solution.

## 1. Introduction

The transition metal coordination compounds have been of great interest over the past decades because of their interesting and diverse structural features and promising applications in different fields of industry. Such compounds can be used in various areas as materials that exhibit desired catalytic, ion-exchange, gas storage, pharmaceutical, luminescence, optical, electronic, or magnetic properties [1,2,3,4,5,6,7,8,9]. Electron-withdrawing metal ions, such as mercury(II), can change and tune the fluorescent properties of the organic ligands used in a design of coordination compounds [8,9,10,11,12,13]. Flexible coordination geometry of the mercury(II) ion, varying from tetrahedral to octahedral, enables the formation of different architectures with different properties [2,11,12,14,15,16,17,18,19]. By changing additional parameters such as the nature of the ligands, type of the counter ions, selection of solvents, metal-to-ligand ratios, temperature, pH etc., it is possible to impact the crystal structures and tune the desired properties of the coordination compounds [20,21,22,23,24]. Deeper understanding of the above-mentioned parameters is crucial for better prediction of the properties and structures of the solid-state materials. Approaches to crystal growth and design, together with the study and understanding of intermolecular interactions, represent one of the main challenges in the field of crystal engineering [25,26,27,28,29].

Pyridine-2-carboxylic acid (picolinic acid, picH) is a very popular and widely used chelating ligand that is able to coordinate to metal ions in an *N*,*O*-bidentate fashion via pyridine N and carboxylate O atoms. The coordination potential of picolinate ion (pic^−^) can be used for designing different metal-organic architectures [30,31,32,33,34,35,36,37,38]. Indeed, picolinate complexes of almost every metal from the periodic system have been prepared and structurally characterized by now [39], including mercury(II). Monomeric [17] and polymeric [40,41,42] mercury(II) complexes with picolinate, but also with picolinate derivatives (e.g., methyl, ethyl, *n*-propyl and *i*-propyl esters [41,43] and ethyl ester of 6-methylpicolinate [41]), are known from the literature. The mercury(II) complex [HgI(pic)(picH)], containing iodide and both picolinate ion and picolinic acid molecule [17], has been prepared previously. As it became evident that slight changes in the preparation procedures (e.g., metal-to-ligand ratio, solvent choice, pH of the solution, temperature) of mercury(II) complexes with picolinate and its derivatives lead to complexes of various compositions and dimensionalities, the possibility of obtaining new picolinate mercury(II) complexes under defined experimental conditions needed to be examined further. A goal was to correlate the experimental conditions used during syntheses with the composition and dimensionality of the complexes.

A one-dimensional (1-D) mercury(II) coordination polymer with picolinate and chloride ion, namely *poly*-chloro(pyridine-2-carboxylato-*N*,*O*,*O*′)mercury(II), {[HgCl(pic)]}*_n_* (**1**), and two isostructural mononuclear mercury(II) complexes with halide ions (chloride or bromide) and both picolinate ion and picolinic acid molecule, namely chloro(pyridine-2-carboxylato-*N*,*O*)(pyridine-2-carboxylic acid-*N*,*O*)mercury(II) [HgCl(pic)(picH)] (**2**) and bromo(pyridine-2-carboxylato-*N*,*O*)(pyridine-2-carboxylic acid-*N*,*O*)mercury(II), [HgBr(pic)(picH)] (**3**), have been prepared under defined experimental conditions.

## 2. Results and Discussion

### 2.1. Syntheses Aspects

**Scheme 1:**
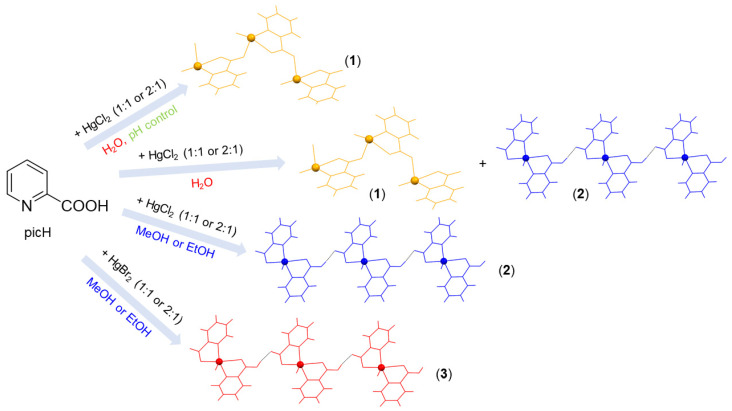
Preparation of {[HgCl(pic)]}*_n_* (**1**), [HgCl(pic)(picH)] (**2**) and [HgBr(pic)(picH)] (**3**) by reactions of pyridine-2-carboxylic acid with mercury(II) chloride and mercury(II) bromide, respectively.

The reactions of mercury(II) chloride and bromide with picolinic acid were studied in aqueous, methanol and ethanol solutions (using two different metal-to-ligand ratios, 1:1 and 1:2) to see how the solvent change and metal-to-ligand ratio would affect the obtained complexes (Scheme 1).

The coordination polymer **1** was obtained in an aqueous solution by using a metal-to-ligand ratio of 1:1 or 1:2 and adjusting the pH of the reaction mixture until precipitation of the compound was completed, while mononuclear complexes **2** and **3** were obtained in methanol or ethanol solutions by using a metal-to-ligand ratio of 1:1 or 1:2. Reactions of HgCl_2_ with picH performed in water solution without increasing the pH value always led to the mixture of polymeric and mononuclear complexes. Regardless of the metal-to-ligand ratios (1:1 or 1:2), the mononuclear complex **3** was always obtained. Reactions were performed only in methanol and ethanol due to the very poor solubility of the mercury(II) bromide in water. The presence of water and a slight increase of the pH value favors the formation of the coordination polymer **1**.

### 2.2. Crystal Structures

**Figure 1:**
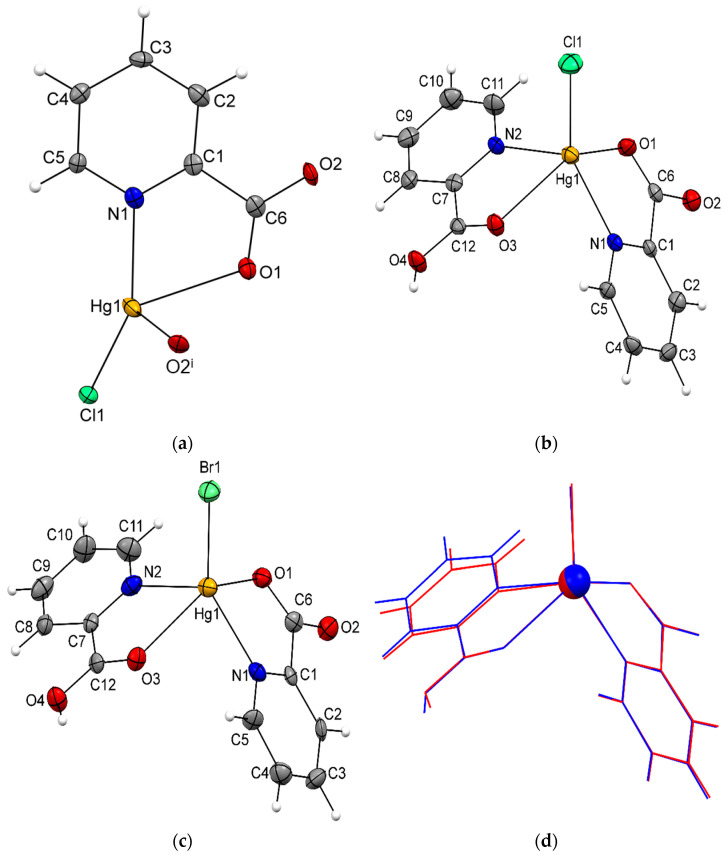
ORTEP-style plots of {[HgCl(pic)]}*_n_* (**1**) (**a**), [HgCl(pic)(picH)] (**2**) (**b**), and [HgBr(pic)(picH)] (**3**) (**c**), with the atomic numbering schemes. Thermal ellipsoids are drawn at the 40% probability level at 170(2) K, and hydrogen atoms are shown as spheres of arbitrary radii (symmetry code (i): *x*, −*y* + 3/2, *z* + 1/2). Overlay (RMS value of 0.0556 Å) (**d**) of molecules of [HgCl(pic)(picH)] (**2**) (blue) and [HgBr(pic)(picH)] (**3**) (red). The Hg, N, O, and halide atoms were chosen for the overlay.

**Figure 2:**
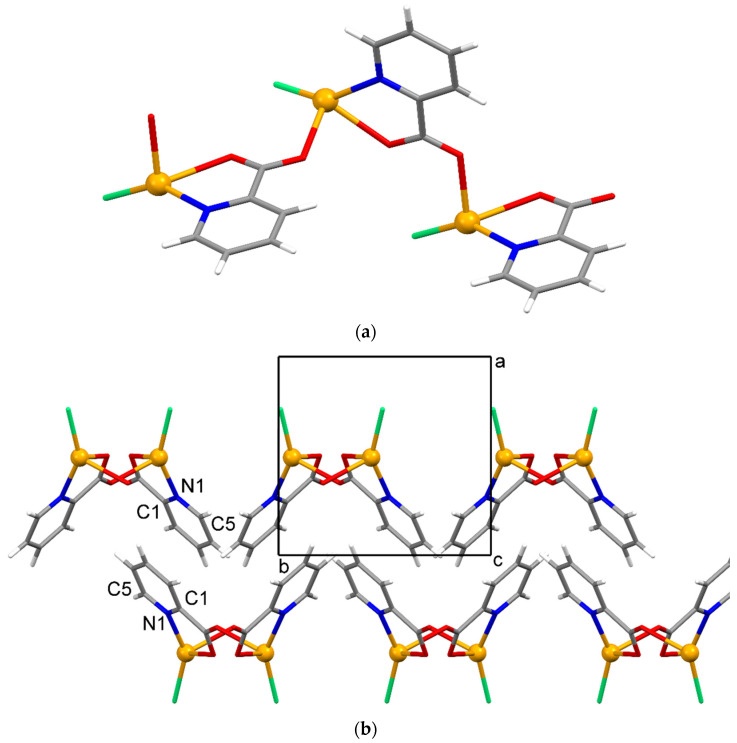
The infinite one-dimensional polymeric zigzag chain of {[HgCl(pic)]}*_n_* (**1**) extending along the [0 0 1] direction (**a**). A fragment of the structure of {[HgCl(pic)]}*_n_* (**1**) viewed down the [0 0 1] direction. The polymeric chains, represented as monomeric molecules in this projection, are connected along the [0 1 0] direction by π–π interactions between symmetry-related picolinate pyridine rings (N1/C1–C5); the selected atoms of the pyridine rings in the interactions are labeled (**b**).

The asymmetric unit of {[HgCl(pic)]}*_n_* (**1**) consists of a mercury(II) ion, a chloride ion and a picolinate ion. The mercury(II) ion is four-coordinated with the chloride Cl1 ion, pyridine N1, and carboxylate O1 and O2^i^ atoms (symmetry code (i) *x*, –*y* + 3/2, *z* + 1/2), resulting in a coordination environment that can be described as a highly distorted tetrahedron (*τ*_4_ value [44] of 0.67; Figure 1a and Table S1 in the Supplementary Materials). The adjacent mercury(II) ions in **1** are bridged by an *N*,*O*,*O*′-picolinate ion, thus forming an infinite 1-D polymeric zigzag chain extending in the [0 0 1] direction (Figure 2a). Each *N*,*O*,*O*′-picolinate ion acts as a chelating and bridging ligand (via pyridine N1 and carbxylate O1 atoms, forming a five-membered chelate ring Hg1/O1/C6/C1/N1, and via carboxylate O2^i^ atom) between two neighboring mercury(II) ions (Figure 2a).

The asymmetric units of [HgCl(pic)(picH)] (**2**) and [HgBr(pic)(picH)] (**3**) consist of a mercury(II) ion, a halide ion (chloride in **2** and bromide in **3**), a picolinate ion, and a picolinic acid molecule (Figure 1b,c). Compounds **2** and **3** are isostructural, as can be seen from the overlay of the respective molecules of **2** and **3** (Figure 1d). Moreover, both compounds **2** and **3** crystallize in the same space group (*P*2_1_/*c*) and with the similar unit cell parameters.

The mercury(II) ion in **2** and **3** is five-coordinated with pyridine N1 and N2 atoms and carboxylate O1 and O3 atoms, which are all situated in a basal plane of a square pyramid, whilst chloride (in **2**) or bromide (in **3**) ions are placed in the apical position (Figure 1b,c, Table S1 in the Supplementary Materials). The pyridine N1 and N2 atoms are in the *trans* position (bond angles N1−Hg1−N2 = 115.3(2)° (in **2**) and 114.2(4)° (in **3**)); and also, the carboxylate O1 and O3 atoms (bond angles O1−Hg1−O3 = 135.7(2)° (in **2**) and 136.7(3)° (in **3**)). Both the picolinate ion and the picolinic acid molecule are bound to the mercury(II) ion as chelating ligands, in the *N*,*O*-bidentate mode (via pyridine N and carboxylate O atoms) in **2** and **3**, forming two five-membered chelate rings (Hg1/O1/C6/C1/N1 and Hg1/O3/C12/C7/N2) per molecule. The *τ* values [45] (0.08 for **2** and 0.02 for **3**) suggest that the mercury(II) coordination environment in both **2** and **3** can be best described as a square pyramid. The mercury(II) ion is significantly displaced from the center of the basal plane toward the halide ion in both **2** and **3**, with a maximum out-of-plane deviation of 1.081(2) Å (in **2**) and 1.079(6) Å (in **3**).

The highly distorted tetrahedral coordination environment in **1** can be also seen in a wide range of bond angles around the mercury(II) ion (72.6(2)°–147.0(1)°; Table S1 in the Supplementary Materials). The square-pyramidal coordination environments around the mercury(II) ions in **2** and **3** are also highly distorted, as the angles for the *trans* (115.3(2)°–135.7(2)° in **2** and 114.2(4)°–136.7(3)° in **3**) and *cis* (68.4(2)°–140.6(1)° in **2** and 68.7(4)°–136.7(3)° in **3**) pairs of the ligating atoms indicate (Table S1 in the Supplementary Materials). The reason for such a distortion in **2** and **3** is probably the *N*,*O*-bidentate binding of both picolinate ion and picolinic acid molecule and the formation of two five-membered chelate rings per a single molecule, with very small binding angles N1–Hg1–O1 (71.7(2)° in **2** and 68.7(4)° in **3**), N2–Hg1–O3 (68.4(2)° in **2** and 71.6(4)° in **3**; Table S1 in the Supplementary Materials). The Hg–N and Hg–O (pyridine N and carboxylate O atoms) bond lengths in **1**–**3** are comparable to those seen in the related mercury(II) complexes containing picolinate ions [17,40,41,42] and the mentioned picolinate derivatives [41,43].

A fairly similar 1-D mercury(II) coordination polymer with picolinate, {[Hg(pic)_2_]}*_n_,* is known from the literature [40,41]. Although picolinate ions in {[Hg(pic)_2_]}*_n_* act in the same way (chelating and bridging ligands) as in **1**, there are no halide ions in {[Hg(pic)_2_]}*_n_*, and two picolinate ions bridge two neighboring mercury(II) ions (as opposed to one bridging picolinate in **1**), leading to an octahedral coordination of mercury(II) ions in {[Hg(pic)_2_]}*_n_*[40,41]. The complexes **2** and **3** are very similar to the known [HgI(pic)(picH)], but they are not isostructural, as the unit cell parameters and the space group of [HgI(pic)(picH)] differs [17]. However, while mercury(II) ions in **2** and **3** exhibit a distorted square-pyramidal coordination, mercury(II) ion in [HgI(pic)(picH)] shows a distorted trigonal-bipyramidal coordination [17].

The crystal structure of **1** features no classical hydrogen bonds, while only strong intermolecular O–H⋅⋅⋅O hydrogen bonds are present in the crystal structures of **2** and **3** (Table S2 in the Supplementary Materials). The π–π interactions [46] are present in all three structures. The described polymeric chains of **1**, running along the [0 0 1] direction, are connected along the [0 1 0] direction by π–π interactions between symmetry-related picolinate pyridine rings [a *Cg*1⋅⋅⋅*Cg*1(−*x*, −*y*, −*z*) distance = 3.492(4) Å, a dihedral angle between the planes = 0.0(3)°, and a slippage = 1.323 Å, where *Cg*1 is the centroid of the picolinate pyridine ring N1/C1–C5] (Figure 2b).

**Figure 3:**
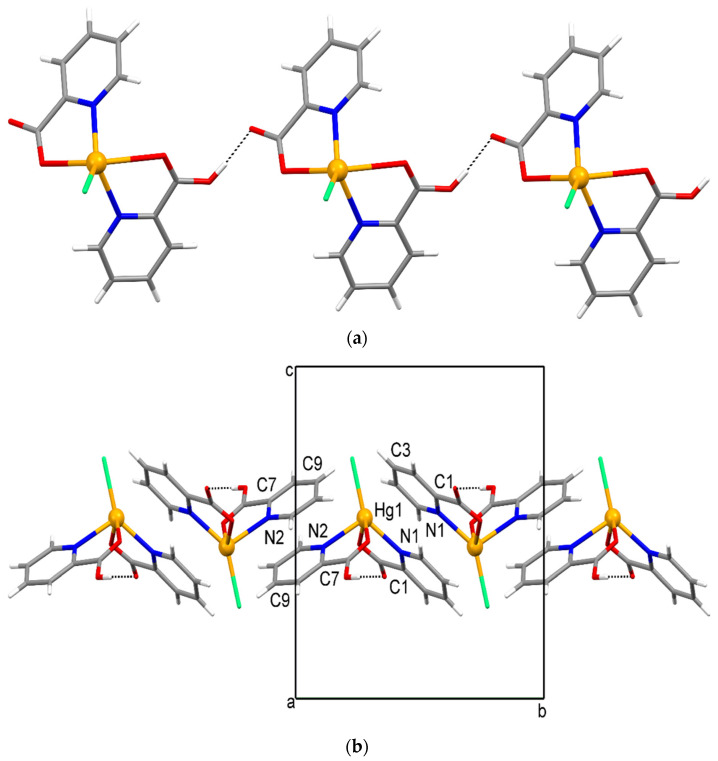
A hydrogen-bonded chain in the structure of [HgCl(pic)(picH)] (**2**). The [HgCl(pic)(picH)] molecules are connected by the intermolecular O–H⋅⋅⋅O hydrogen bonds (represented by the dotted lines) along the [1 0 0] direction, giving rise to a hydrogen-bond chain motif C11(8) (**a**). A fragment of the structure of [HgCl(pic)(picH)] (**2**) viewed down the [1 0 0] direction. The hydrogen-bonded chains of [HgCl(pic)(picH)], represented as monomeric molecules in this projection, are connected along the [0 1 0] direction by π–π interactions between symmetry-related picolinic acid pyridine rings (N2/C7–C11) and between picolinate pyridine rings (N1/C1–C5) and chelate rings (Hg1/O1/C6/C1/N1); the selected atoms of the pyridine rings in the interactions are labeled (**b**).

The [HgCl(pic)(picH)] molecules in **2** and [HgBr(pic)(picH)] in **3** are connected by the intermolecular O–H⋅⋅⋅O hydrogen bonds along the [1 0 0] direction, giving rise to a hydrogen-bond chain motif *C*^1^_1_(8) (Figure 3a). These hydrogen-bonded chains of [HgCl(pic)(picH)] in **2** and [HgBr(pic)(picH)] in **3**, respectively, are connected along the [0 1 0] direction by π–π interactions between symmetry-related picolinic acid pyridine rings [a *Cg*2⋅⋅⋅*Cg*2(2–*x*, –*y*, 1–*z* in **2** and –*x*, 1–*y*, –*z* in **3**) distance = 3.795(4) Å (in **2**) and 3.957(8) Å (in **3**), a dihedral angle between the planes = 0.0(3)° (in **2**) and 0.0(7)° (in **3**) and a slippage = 1.681 Å (in **2**) and 1.951 Å (in **3**), where *Cg*2 is the centroid of the picolinic acid pyridine ring N2/C7–C11] and between picolinate pyridine rings and chelate rings [a *Cg*3⋅⋅⋅*Cg*4(1–*x*, 1–*y*, 1–*z* in **2** and 1–*x*, –*y*, –*z* in **3**) distance = 3.588(3) Å (in **2**) and 3.656(7) Å (in **3**), a dihedral angle between the planes = 3.7(3)° (in **2**) and 3.9(6)° (in **3**) and a slippage = 1.107 Å (in **2**) and 1.192 Å (in **3**), where *Cg*3 is the centroid of the picolinate pyridine ring N1/C1–C5 and *Cg*4 is the centroid of the chelate ring Hg1/O1/C6/C1/N1)] (Figure 3b).

**Figure 4:**
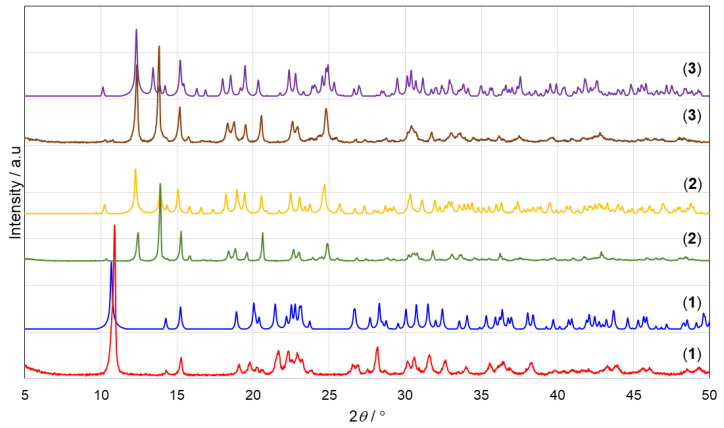
Overlay of experimental (red, green, brown) and calculated (blue, yellow, violet) PXRD traces for {[HgCl(pic)]}*_n_* (**1**), [HgCl(pic)(picH)] (**2**) and [HgBr(pic)(picH)] (**3**).

PXRD was used to confirm the phase purity and bulk composition of compounds **1**–**3** (Figure 4). The powder diffraction traces (bulk sample) of all three compounds are consistent with the traces calculated from the single crystal diffraction data, indicating pure phases of compounds **1**–**3**. The almost identical powder patterns of isostructural compounds **2** and **3** are consistent with their crystal structures.

### 2.3. Spectroscopic Studies (FT-IR, NMR)

The recorded IR spectra of coordination polymer **1** and mononuclear compounds **2** and **3** (Figure S1 in the Supplementary Materials) are in a good agreement with their crystal structures. Very broad bands in the spectra of **2** and **3** centered around 3400 cm^−1^ can be attributed to the hydrogen-bonded (O–H⋅⋅⋅O) carboxylic groups [47,48]. The broad bands in the IR spectrum of free picH centered around 2600 and 2100 cm^−1^ are connected with the O–H⋯N intermolecular hydrogen bonding. The disappearing of the above-mentioned bands from the IR spectra of the metal complexes suggests a coordination of the N atom to the mercury(II) ion [48,49]. The characteristic *ν*(C=O) bands of the carboxylic group at 1718 cm^−1^ in the IR spectra of **2** and **3** are connected with the existence of neutral picolinic acid molecules [47,48]. On the other hand, the absence of the bands around 3400 and 1700 cm^−1^ in the IR spectrum of **1** indicates a complete deprotonation of the carboxylic group [41,48,49,50,51]. The IR spectra of the metal complexes show strong absorptions of the antisymmetric *ν*_as_(COO^−^) and symmetric *ν*_s_(COO^−^) stretching vibrations of the carboxylate ligands in the 1650−1590 cm^−1^ and 1380−1350 cm^−1^ regions.

**Scheme 2:**
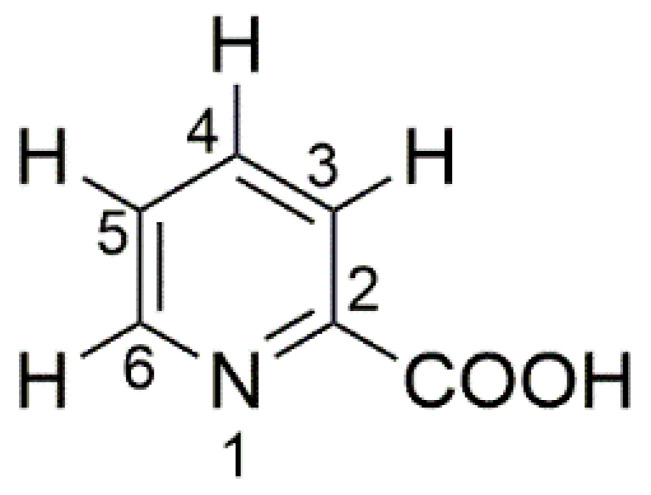
Numeration of the atoms in picH.

The ^13^C NMR data for picH and compounds **1**–**3** are given in Table 1. The numeration of the atoms is consistent with the IUPAC nomenclature (Scheme 2). The complexation effects due to the mercury(II)-induced electronic redistribution throughout the coordinated ligands are well-known from the literature [18,19,41]. The NMR data of **1**–**3** show small but significant complexation effects. The greatest changes upon complexation with mercury(II) are observed for the coordination polymer **1**. In the ^13^C NMR spectra of all complexes, the greatest change (deshielding effect, 4.27–1.38 ppm) upon complexation with mercury(II) was observed for the chemical shift of the C-4 atoms. The largest shielding effects (2.89–1.66 ppm) were observed for the carboxylate carbon atoms, being only two bonds away from the coordinated mercury(II) ion. The shielding effect is consistent with a reduction of the π–electron density of the C=O bond due to the carboxylic/carboxylate coordination to mercury. Small but significant complexation effects observed in the NMR spectra of prepared complexes are in agreement with the existence of mononuclear (**2** and **3**) and polymeric (**1**) species in the DMSO solutions.

### 2.4. Thermal Analysis (TGA/DSC)

The thermal behavior of compounds **1**–**3** has been investigated by simultaneous TGA/DSC analysis from room temperature up to 450 °C in flowing nitrogen (Figure S2 in the Supplementary Materials).

Continuous thermal degradation of the compounds was observed. Total weight losses of 87.28% (**1**), 89.42% (**2**), and 89.05% (**3**) were observed at 450 °C. Thermal degradation is characterized with two (**1**) and one (**2** and **3**) signals on the DSC curves: 203 and 206 °C, 206 and 204 °C respectively. According to the literature [51,52,53], the above-mentioned signals probably correspond to the decarboxylation of picH.

## 3. Experimental Section

### 3.1. Materials and Physical Measurements

All commercially available chemicals were of reagent grade and were used as received without further purification. CHN elemental analyses were carried out with a Perkin-Elmer 2400 Series II CHNS analyzer in Analytical Services Laboratories of the Ruđer Bošković Institute, Zagreb, Croatia. The mercury content in the complexes was determined by complexometric titration with sodium diethyldithiocarbamate of the solution obtained after decomposition of the compounds in *aqua regia* [54]. The pH values were measured by a Mettler Toledo MP220 Basic pH/mV/°C Meter.

IR spectra were obtained from KBr pellets in the range 4000–500 cm^−1^ on a Perkin-Elmer Spectrum Two FT-IR spectrometer. The one-dimensional homonuclear ^1^H and ^13^C NMR spectra were recorded with a Bruker AV 600 spectrometer, operating at 600.133 MHz for the ^1^H nucleus and 150.917 MHz for the ^13^C nucleus. The samples were dissolved in DMSO-*d*_6_ in 5 mm NMR tubes. Chemical shifts, in ppm, are referred to TMS as internal standard.

Thermogravimetric analysis was performed using a simultaneous TGA-DSC analyzer Mettler-Toledo TGA/DSC 3+. The samples of compounds **1**–**3** were placed in alumina pans (70 μL) and heated in flowing nitrogen (50 mL min^−1^) from room temperature up to 450 °C at a rate of 10 °C min^−1^. Data collection and analysis were performed using the program package STARe Software 15.01 MettlerToledo GmbH, 2015.

Powder X-ray diffraction experiments (PXRD) were measured on a Malvern Panalytical Aeris XRD diffractometer with CuKα (1.5406 Å) radiation, Ni filter, and solid-state PIXcel3D-Medipix3 detector. Samples were prepared as a thin layer on a silicon zero-background plate. Data were collected in the 2*θ* range from 5° to 50° with a step size of 0.02173°, scan rate 10 s/°, ¼ inch divergence slit, and 13 mm beam mask.

### 3.2. Syntheses of the Compounds

#### 3.2.1. Synthesis of {[HgCl(pic)]}_n_ (**1**)

A solution of picH (0.23 g, 1.87 mmol) in 10 mL H_2_O was added dropwise to a solution of HgCl_2_ (0.50 g, 1.84 mmol) in 20 mL H_2_O. An aqueous solution of NaOAc (5%) was added to the reaction mixture until precipitation of the compound was completed (increasing the pH from 2.38 to 2.76). The colorless product was filtered off and washed with a small amount of water and dried in air. Yield: 62% (based on HgCl_2_). Anal. Calcd for HgClC_6_H_4_NO_5_: C, 20.12; H, 1.33; N, 3.91; Hg, 56.01. Found: C, 20.10; H, 1.38; N 3.91; Hg, 56.97.

FT-IR (KBr, cm^−1^): 1621(w), 1608(m), 1583(s), 1557(vs), 1382(m), 1367(s), 1301(w), 1260(m), 1236(w), 1169(w), 1091(w), 1055(m), 1025(m), 914(w), 834(m), 761(vs), 693(vs), 647(m), 534(w).

^1^H NMR (DMSO-*d*_6_, ppm): 8.83 (d, *J*_HH_ = 5.207 Hz, H-6), 8.43 (d, *J*_HH_ = 7782 Hz, H-3), 8.27 (t, *J*_HH_ = 7599 Hz, H-4), 7,92 (t, *J*_HH_ = 6822 Hz, H-5).

Preparation of single crystals of {[HgCl(pic)]}*_n_* (**1**).

A solution of picH (0.12 g, 0.97 mmol) in 10 mL H_2_O was added dropwise to a solution of HgCl_2_ (0.30 g, 0.94 mmol) in 15 mL H_2_O. Small portions of colorless crystals of **1**, suitable for single-crystal X-ray structure determination, were formed in a few hours. The mixture of **1** and **2** was isolated by allowing the mother liquor to stand for a few days.

#### 3.2.2. Synthesis of [HgCl(pic)(picH)] (**2**)

A solution of picH (0.46 g, 3.74 mmol) in 20 mL MeOH was added dropwise to a solution of HgCl_2_ (0.50 g, 1.84 mmol) in 10 mL MeOH. Colorless crystals suitable for single-crystal X-ray structure determination were formed in a few days. The colorless crystals were filtered off, washed with MeOH, and dried in air. Yield: 48% (based on HgCl_2_). Anal. Calc. for HgClC_12_H_9_N_2_O_4_: C 29.95, H 1.89, N 5.82, Hg 41.68. Found: C 29.80, H 2.07, N 5.79, Hg 41.54%.

FT-IR (KBr, cm^−1^): 3473(m), 1718(m), 1656(s), 1642(s), 1625(s), 1592(vs), 1569(s), 1440(m), 1383(vs), 1295(s), 1259(m), 1238(m), 1156(m), 1050(m), 1018(m), 998(m), 838(m), 752(s), 698(s), 688(s), 641(w), 624(w), 507(w).

^1^H NMR (DMSO-*d*_6_, ppm): 8.77 (d, *J*_HH_ = 5.008 Hz, H-6), 8.26 (d, *J*_HH_ = 7.722 Hz, H-3), 8.14 (t, *J*_HH_ = 7.593 Hz, H-4), 7,78 (t, *J*_HH_ = 6.379 Hz, H-5).

#### 3.2.3. Synthesis of [HgBr(pic)(picH)] (**3**)

A solution of picH (0.35 g, 2.84 mmol) in 15 mL MeOH was added dropwise to a solution of HgBr_2_ (0.50 g, 1.39 mmol) in 15 mL MeOH. Colorless crystals suitable for single-crystal X-ray structure determination were formed in a few days. The colorless crystals were filtered off, washed with MeOH, and dried in air. Yield: 49% (based on HgBr_2_). Anal. Calc. for HgBrC_12_H_9_N_2_O_4_: C 27.39, H 1.73, N 5.33, Hg 38.16. Found: C 27.29, H 1.71, N 5.47, Hg 37.98%.

FT-IR (KBr, cm^−1^): 3472(m), 3075(m), 1718(m), 1656(s), 1634(vs), 1618(s), 1593(vs), 1569(s), 1520(w), 1446(w), 1383(vs), 1295(s), 1258(m), 1242(m), 1157(w), 1090 (m), 1051(w), 1018(w), 998(m), 837(m), 753(s), 699(s), 688(s), 641(w), 624(w), 514(w).

^1^H NMR (DMSO-*d*_6_, ppm): 8.73 (d, *J*_HH_ = 4279 Hz, H-6), 8.23 (d, *J*_HH_ = 7.756 Hz, H-3), 8.10 (t, *J*_HH_ = 7.633 Hz, H-4), 7,74 (t, *J*_HH_ = 8.266 Hz, H-5).

### 3.3. X-ray Crystallographic Analysis

Suitable single crystals of **1**–**3** were selected and mounted in Paratone-N oil onto cryo-loops. Data collection was carried out on an XtaLAB Synergy-S Dualflex diffractometer with PhotonJet (Mo) microfocus X-ray source and HyPix-6000HE hybrid photon counting (HPC) X-ray area detector, using graphite monochromated MoKα (*λ* = 0.71073 Å) radiation at low temperature (170(2) K) and by applying the CrysAlisPro Software system [55]. Data reduction and cell refinement were performed by the CrysAlisPro Software system [55]. The structures were solved by SHELXT [56] and refined by SHELXL-2018/3 [57]. The refinement procedure was done by the full-matrix least-squares methods based on *F*^2^ values against all reflections. The figures were made with MERCURY (Version 2020.2.0) [58]. The crystallographic data for **1**–**3** are summarized in Table 2.

## 4. Conclusions

The neighboring mercury(II) ions are bridged by an *N*,*O*,*O*′-picolinate ion into a polymeric chain of **1**, while both complexes **2** and **3** are mononuclear, containing the respective halide ion and *N*,*O*-bidentate picolinate ion and picolinic acid molecule. It is possible to control both the composition and dimensionality of the mercury(II) complexes formed in the system containing mercury(II), picolinic acid, halide ions, and a solvent. This control was achieved by varying the solvent type and metal-to-ligand ratio in this system. The IR, ^1^H, and ^13^C NMR data correlate well with the solid-state structures of **1**, **2**, and **3**. According to the NMR data, there is no decomposition of **1**, **2,** and **3** in the respective DMSO solutions and **1** remains polymeric, while **2** and **3** remain mononuclear in the solutions. The observed differences in the chemical shifts were ascribed to complexation effects and were reliable enough to distinguish between polymeric (**1**) and monomeric (**2** and **3**) compounds.

## Figures and Tables

**Table 1:** ^13^C NMR chemical shifts (*δ*) ^a^ in ppm of picH, {[HgCl(pic)]}*_n_* (**1**), [HgCl(pic)(picH)] (**2**), and [HgBr(pic)(picH)] (**3**).

Compound	C-2	C-3	C-4	C-5	C-6	COOH
picH	148.41	124.76	137.60	127.17	149,49	166.27
**1**	149.30	125.62	141.87	127.35	148.79	163.38
**2**	148.79	125.24	139.88	127.30	149.11	164.78
**3**	148.48	124.57	138.98	126.66	148.51	164.61

^a^ Recorded in DMSO-*d*_6_ solutions. Referred to TMS.

**Table 2:** Crystallographic data for {[HgCl(pic)]}*_n_* (**1**), [HgCl(pic)(picH)] (**2**) and [HgBr(pic)(picH)] (**3**).

Compound	1	2	3
Formula	C_6_H_4_HgClNO_2_	C_12_H_9_HgClN_2_O_4_	C_12_H_9_HgBrN_2_O_4_
*M*_r_	358.14	481.25	525.71
Crystal system, space group	monoclinic, *P*2_1_/*c* (No. 14)	monoclinic, *P*2_1_/*c* (No. 14)	monoclinic, *P*2_1_/*c* (No. 14)
*a* (Å)	9.0131(6)	9.1384(5)	9.1223(4)
*b* (Å)	8.8569(4)	11.7625(8)	11.6466(6)
*c* (Å)	9.4804(6)	12.7526(7)	13.1976(6)
*β* (°)	113.292(7)	92.798(5)	92.073(4)
*V* (Å^3^)	695.12(8)	1369.15(14)	1401.25(11)
*Z*	4	4	4
*D*_calc_ (g cm^−3^)	3.422	2.335	2.492
*μ* (mm^−1^)	22.462	11.450	13.848
*R* [*I* ≥ 2*σ*(*I*)]	0.0265	0.0325	0.0575
w*R* [all data]	0.0652	0.0804	0.1896

## Supplementary Materials

Table S1: Selected bond lengths (Å) and angles (°) for {[HgCl(pic)]}*_n_* (**1**), [HgCl(pic)(picH)] (**2**) and [HgBr(pic)(picH)] (**3**), Table S2: The hydrogen bond geometry for {[HgCl(pic)]}*_n_* (**1**), [HgCl(pic)(picH)] (**2**) and [HgBr(pic)(picH)] (**3**), Figure S1: IR spectra of {[HgCl(pic)]}*_n_* (**1**), [HgCl(pic)(picH)] (**2**) and [HgBr(pic)(picH)] (**3**), Figure S2: TGA/DSC curves of {[HgCl(pic)]}*_n_* (**1**), [HgCl(pic)(picH)] (**2**) and [HgBr(pic)(picH)] (**3**). Deposition numbers 2092545 (for **1**), 2092546 (for **2**) and 2092547 (for **3**) contain the supplementary crystallographic data for this paper. These data are provided free of charge by the joint Cambridge Crystallographic Data Centre and Fachinformationszentrum Karlsruhe Access Structures service www.ccdc.cam.ac.uk/structures.

## Sample Availability

Samples of the compounds **1**–**3** are available from the authors.
