# Supplementary material for: The Solvent Effect on Composition and Dimensionality of Mercury(II) Complexes with Picolinic Acid
Source: Molecules. 2021 Aug 18;26(16):5002. doi: 10.3390/molecules26165002 (PMC8400068; doi:10.3390/molecules26165002)

checkCIF/PLATON report

Structure factors have been supplied for datablock(s) 1

THIS REPORT IS FOR GUIDANCE ONLY. IF USED AS PART OF A REVIEW PROCEDURE FOR PUBLICATION, IT SHOULD NOT REPLACE THE EXPERTISE OF AN EXPERIENCED CRYSTALLOGRAPHIC REFEREE.

No syntax errors found. CIF dictionary Interpreting this report

Datablock: 1

```
Bond precision:  C-C = 0.0086 A          Wavelength=0.71073

Cell:            a=9.0131(6)     b=8.8569(4)         c=9.4804(6)
                 alpha=90        beta=113.292(7)     gamma=90
Temperature:     170 K

                Calculated                Reported
Volume          695.12(8)                 695.12(8)
Space group     P 21/c                    P 21/c
Hall group      -P 2ybc                   -P 2ybc
Moiety formula  C6 H4 Cl Hg N O2          ?
Sum formula     C6 H4 Cl Hg N O2          C6 H4 Cl Hg N O2
Mr              358.14                    358.14
Dx,g cm-3       3.422                     3.422
Z               4                         4
Mu (mm-1)       22.462                    22.462
F000            640.0                     640.0
F000'           632.13
h,k,lmax        10,10,11                  10,10,11
Nref            1222                      1219
Tmin,Tmax                                 0.341,1.000
Tmin'

Correction method= # Reported T Limits: Tmin=0.341 Tmax=1.000
AbsCorr = MULTI-SCAN

Data completeness= 0.998          Theta(max)= 24.996

R(reflections)= 0.0265( 1155)     wR2(reflections)= 0.0652( 1219)

S = 1.089                  Npar= 100
```

The following ALERTS were generated. Each ALERT has the format
test-name_ALERT_alert-type_alert-level.
Click on the hyperlinks for more details of the test.

Alert level C

```
PLAT053_ALERT_1_C Minimum Crystal Dimension Missing (or Error) ...     Please Check
PLAT054_ALERT_1_C Medium  Crystal Dimension Missing (or Error) ...     Please Check
PLAT055_ALERT_1_C Maximum Crystal Dimension Missing (or Error) ...     Please Check
PLAT232_ALERT_2_C Hirshfeld Test Diff (M-X)  Hg1      --O2_b     .        6.0 s.u.
PLAT342_ALERT_3_C Low Bond Precision on  C-C Bonds ...............     0.0086 Ang.
PLAT369_ALERT_2_C Long   C(sp2)-C(sp2) Bond  C1       - C6       .       1.54 Ang.
PLAT911_ALERT_3_C Missing FCF Refl Between Thmin & STh/L=    0.595          2 Report
PLAT976_ALERT_2_C Check Calcd Resid. Dens.  0.93A   From O1              -0.93 eA-3
PLAT977_ALERT_2_C Check Negative Difference Density on H5                -0.40 eA-3
```

Alert level G

```
PLAT004_ALERT_5_G Polymeric Structure Found with Maximum Dimension          1 Info
PLAT432_ALERT_2_G Short Inter X...Y Contact  Cl1      ..C4               3.24 Ang.
                                         1-x,1/2+y,3/2-z  =      2_656 Check
PLAT883_ALERT_1_G No Info/Value for _atom_sites_solution_primary .     Please Do !
PLAT909_ALERT_3_G Percentage of I>2sig(I) Data at Theta(Max) Still        91% Note
PLAT910_ALERT_3_G Missing # of FCF Reflection(s) Below Theta(Min).          1 Note
PLAT913_ALERT_3_G Missing # of Very Strong Reflections in FCF ....          1 Note
PLAT933_ALERT_2_G Number of OMIT Records in Embedded .res File ...          2 Note
PLAT965_ALERT_2_G The SHELXL WEIGHT Optimisation has not Converged     Please Check
PLAT978_ALERT_2_G Number C-C Bonds with Positive Residual Density.          0 Info
```

---

0 **ALERT level A** = Most likely a serious problem - resolve or explain
0 **ALERT level B** = A potentially serious problem, consider carefully
9 **ALERT level C** = Check. Ensure it is not caused by an omission or oversight
9 **ALERT level G** = General information/check it is not something unexpected

4 ALERT type 1 CIF construction/syntax error, inconsistent or missing data
8 ALERT type 2 Indicator that the structure model may be wrong or deficient
5 ALERT type 3 Indicator that the structure quality may be low
0 ALERT type 4 Improvement, methodology, query or suggestion
1 ALERT type 5 Informative message, check

Validation response form

Please find below a validation response form (VRF) that can be filled in and pasted into your CIF.

```
# start Validation Reply Form
_vrf_PLAT053_1
;
PROBLEM: Minimum Crystal Dimension Missing (or Error) ...     Please Check
RESPONSE: ...
;
_vrf_PLAT054_1
;
PROBLEM: Medium  Crystal Dimension Missing (or Error) ...     Please Check
RESPONSE: ...
;
_vrf_PLAT055_1
;
PROBLEM: Maximum Crystal Dimension Missing (or Error) ...     Please Check
RESPONSE: ...
;
_vrf_PLAT232_1
;
PROBLEM: Hirshfeld Test Diff (M-X)  Hg1      --O2_b     .        6.0 s.u.
RESPONSE: ...
```

```
;
_vrf_PLAT342_1
;
PROBLEM: Low Bond Precision on  C-C Bonds ..............     0.0086 Ang.
RESPONSE: ...
;
_vrf_PLAT369_1
;
PROBLEM: Long   C(sp2)-C(sp2) Bond  C1       - C6       .       1.54 Ang.
RESPONSE: ...
;
_vrf_PLAT911_1
;
PROBLEM: Missing FCF Refl Between Thmin & STh/L=    0.595          2 Report
RESPONSE: ...
;
_vrf_PLAT976_1
;
PROBLEM: Check Calcd Resid. Dens.  0.93A   From O1             -0.93 eA-3
RESPONSE: ...
;
_vrf_PLAT977_1
;
PROBLEM: Check Negative Difference Density on H5               -0.40 eA-3
RESPONSE: ...
;
# end Validation Reply Form
```

It is advisable to attempt to resolve as many as possible of the alerts in all categories. Often the minor alerts point to easily fixed oversights, errors and omissions in your CIF or refinement strategy, so attention to these fine details can be worthwhile. In order to resolve some of the more serious problems it may be necessary to carry out additional measurements or structure refinements. However, the purpose of your study may justify the reported deviations and the more serious of these should normally be commented upon in the discussion or experimental section of a paper or in the "special_details" fields of the CIF. checkCIF was carefully designed to identify outliers and unusual parameters, but every test has its limitations and alerts that are not important in a particular case may appear. Conversely, the absence of alerts does not guarantee there are no aspects of the results needing attention. It is up to the individual to critically assess their own results and, if necessary, seek expert advice.

Publication of your CIF in IUCr journals

A basic structural check has been run on your CIF. These basic checks will be run on all CIFs submitted for publication in IUCr journals (*Acta Crystallographica*, *Journal of Applied Crystallography*, *Journal of Synchrotron Radiation*); however, if you intend to submit to *Acta Crystallographica Section C* or *E* or *IUCrData*, you should make sure that full publication checks are run on the final version of your CIF prior to submission.

Publication of your CIF in other journals

Please refer to the *Notes for Authors* of the relevant journal for any special instructions relating to CIF submission.

PLATON version of 03/06/2021; check.def file version of 02/06/2021

Datablock 1 - ellipsoid plot

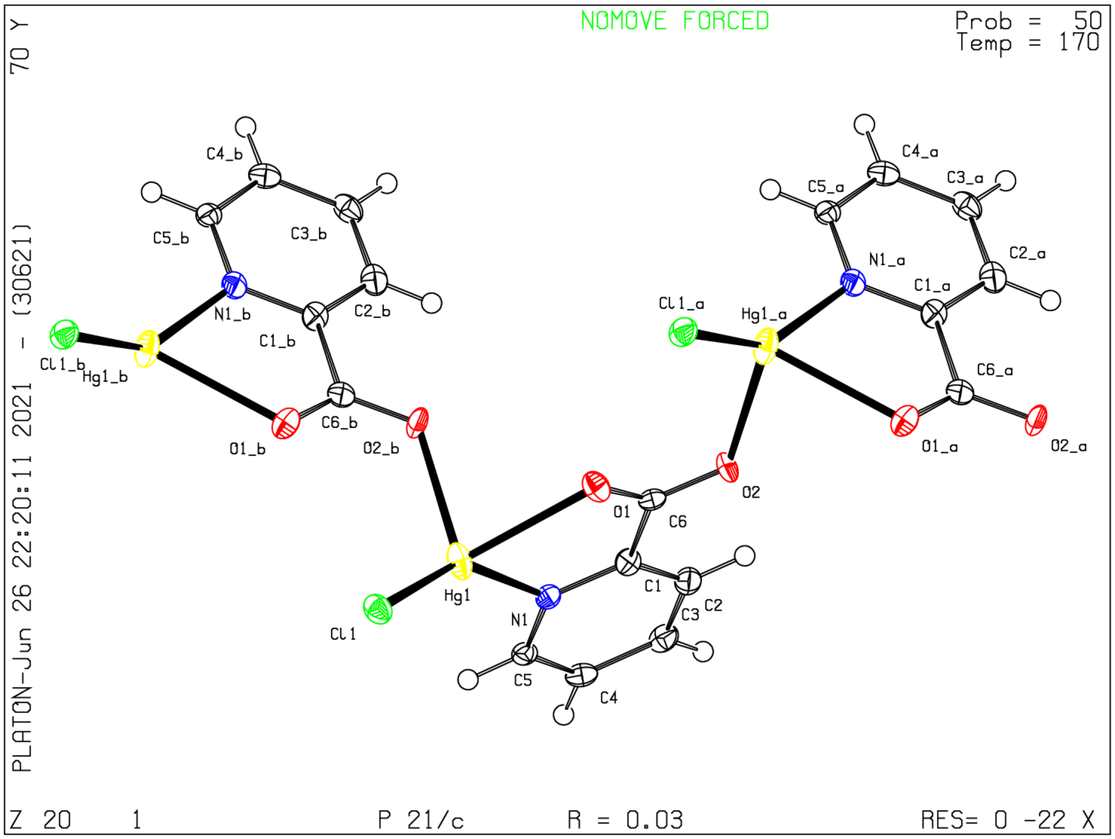

Supplement: Supplementary file 1 [file molecules-26-05002-s001.zip › 1-checkcif.pdf]
