# Supplementary material for: The Solvent Effect on Composition and Dimensionality of Mercury(II) Complexes with Picolinic Acid
Source: Molecules. 2021 Aug 18;26(16):5002. doi: 10.3390/molecules26165002 (PMC8400068; doi:10.3390/molecules26165002)
Supplement: Supplementary file 1 [file molecules-26-05002-s001.zip › supplementary materials.docx]

**Supplementary Materials**

**The solvent effect on composition and dimensionality of mercury(II) complexes with picolinic acid**

**Željka Soldin^a^*, Boris-Marko Kukovec****^b^*, Dubravka Matković-Čalogović^a^, Zora Popović^a^**

*^a^Division of General and Inorganic Chemistry, Department of Chemistry, Faculty of Science, University of Zagreb,* *Horvatovac 102a, HR-10000 Zagreb, Croatia*

*^b^Department of Physical Chemistry, Faculty of Chemistry and Technology, University of Split, Ruđera Boškovića 35, HR-21000 Split, Croatia*

***Corresponding authors:**

Ž. Soldin

e-mail: [zeljka@chem.pmf.hr](mailto:zeljka@chem.pmf.hr)

B.-M. Kukovec

e-mail: [bmkukovec@ktf-split.hr](mailto:bmkukovec@ktf-split.hr)

1. **Crystal structures**

**Table S1.** Selected bond lengths (Å) and angles (˚) for {[HgCl(pic)]}*_n_* (**1**), [HgCl(pic)(picH)] (**2**) and [HgBr(pic)(picH)] (**3**).

| **1** | | **2** | | **3** | |
| --- | --- | --- | --- | --- | --- |
| *Bond lengths* | | | | | |
| Hg1–Cl1 | 2.335(2) | Hg1–Cl1 | 2.352(2) | Hg1–Br1 | 2.461(2) |
| Hg1–N1 | 2.191(5) | Hg1–N1 | 2.212(5) | Hg1–N1 | 2.23(1) |
| Hg1–O1 | 2.411(4) | Hg1–N2 | 2.346(6) | Hg1–N2 | 2.33(1) |
| Hg1–O2^i^ | 2.373(4) | Hg1–O1 | 2.487(4) | Hg1–O1 | 2.47(1) |
|  |  | Hg1–O3 | 2.539(4) | Hg1–O3 | 2.52(1) |
| *Bond angles* | | | | | |
| N1–Hg1–Cl1 | 147.0(1) | N1–Hg1–N2 | 115.3(2) | N1–Hg1–N2 | 114.2(4) |
| N1–Hg1–O2^i^ | 96.7(2) | N1–Hg1–Cl1 | 140.6(1) | N1–Hg1–Br1 | 137.7(3) |
| Cl1–Hg1–O2^i^ | 114.8(1) | N2–Hg1–Cl1 | 104.1(1) | N2–Hg1–Br1 | 108.1(3) |
| N1–Hg1–O1 | 72.6(2) | N1–Hg1–O1 | 71.7(2) | N1–Hg1–O1 | 71.6(4) |
| Cl1–Hg1–O1 | 119.0(1) | N2–Hg1–O1 | 88.6(2) | N2–Hg1–O1 | 87.9(4) |
| O2^i^–Hg1–O1 | 82.7(1) | Cl1–Hg1–O1 | 112.1(1) | Br1–Hg1–O1 | 111.5(3) |
|  |  | N1–Hg1–O3 | 84.3(2) | N1–Hg1–O3 | 85.1(3) |
|  |  | N2–Hg1–O3 | 68.4(2) | N2–Hg1–O3 | 68.7(4) |
|  |  | Cl1–Hg1–O3 | 110.1(1) | Br1–Hg1–O3 | 110.3(3) |
|  |  | O1–Hg1–O3 | 135.7(2) | O1–Hg1–O3 | 136.7(3) |

Symmetry code (i) *x*, –*y*+3/2, *z*+1/2.

**Table S2.** The hydrogen bond geometry for {[HgCl(pic)]}*_n_* (**1**), [HgCl(pic)(picH)] (**2**) and [HgBr(pic)(picH)] (**3**).

| D–H⋅⋅⋅A | *d*(D–H)/Å | *d*(H⋅⋅⋅A)/Å | *d*(D⋅⋅⋅A)/Å | ∠(D–H⋅⋅⋅A)/˚ | Symmetry code on A |
| --- | --- | --- | --- | --- | --- |
| **2** | | | | | |
| O4–H41⋅⋅⋅O2 | 0.84(1) | 1.61(1) | 2.451(6) | 175(8) | *x*+1, *y*, *z* |
| **3** | | | | | |
| O4–H41⋅⋅⋅O2 | 0.84 | 1.61 | 2.43(1) | 163 | *x*–1, *y*, *z* |

1. **IR spectroscopy**

**
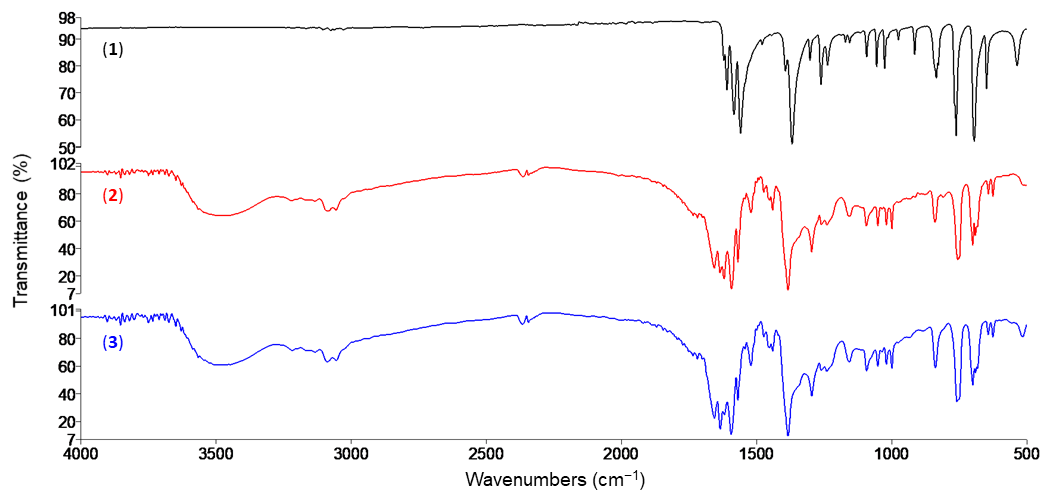
**

**Figure S1.** IR spectra of {[HgCl(pic)]}*_n_* (**1**), [HgCl(pic)(picH)] (**2**) and [HgBr(pic)(picH)] (**3**).

**3. TG/DSC analysis**


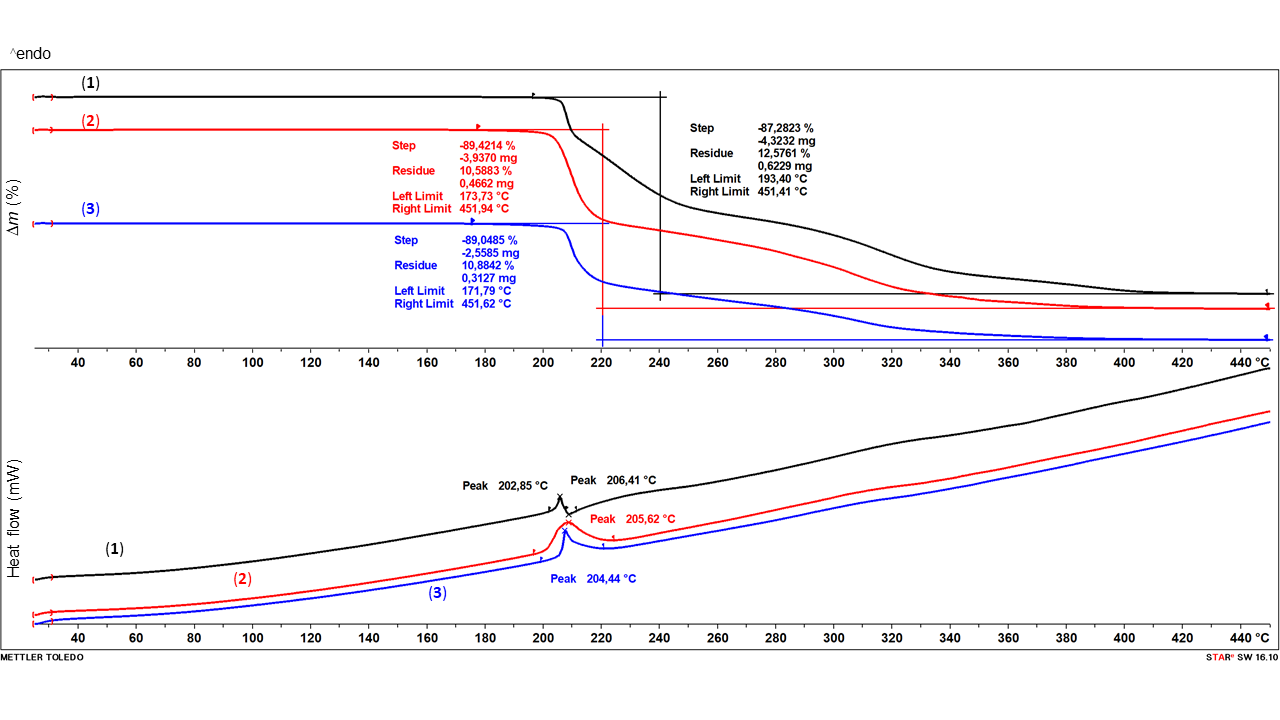


**Figure S2.** TGA/DSC curves of {[HgCl(pic)]}*_n_* (**1**), [HgCl(pic)(picH)] (**2**) and [HgBr(pic)(picH)] (**3**).
