# Supplementary material for: The Solvent Effect on Composition and Dimensionality of Mercury(II) Complexes with Picolinic Acid
Source: Molecules. 2021 Aug 18;26(16):5002. doi: 10.3390/molecules26165002 (PMC8400068; doi:10.3390/molecules26165002)
Supplement: Supplementary file 1 [file molecules-26-05002-s001.zip › supplementary materials.pdf]

Supplementary Materials

The solvent effect on composition and dimensionality of mercury(II) complexes with picolinic acid

Željka Soldin[a]*, Boris-Marko Kukovec[b]*, Dubravka Matković-Čalogović[a], Zora Popović[a]

[a]*Division of General and Inorganic Chemistry, Department of Chemistry, Faculty of Science, University of Zagreb, Horvatovac 102a, HR-10000 Zagreb, Croatia*

[b]*Department of Physical Chemistry, Faculty of Chemistry and Technology, University of Split, Ruđera Boškovića 35, HR-21000 Split, Croatia*

***Corresponding authors:**

Ž. Soldin
e-mail: zeljka@chem.pmf.hr

B.-M. Kukovec
e-mail: bmkukovec@ktf-split.hr

1. Crystal structures

Table S1. Selected bond lengths (Å) and angles (°) for {[HgCl(pic)]}$_n$ (**1**), [HgCl(pic)(picH)] (**2**) and [HgBr(pic)(picH)] (**3**).

**1**		**2**		**3**	
		Bond lengths			
Hg1–Cl1	2.335(2)	Hg1–Cl1	2.352(2)	Hg1–Br1	2.461(2)
Hg1–N1	2.191(5)	Hg1–N1	2.212(5)	Hg1–N1	2.23(1)
Hg1–O1	2.411(4)	Hg1–N2	2.346(6)	Hg1–N2	2.33(1)
Hg1–O2^{i}	2.373(4)	Hg1–O1	2.487(4)	Hg1–O1	2.47(1)
		Hg1–O3	2.539(4)	Hg1–O3	2.52(1)
		Bond angles			
N1–Hg1–Cl1	147.0(1)	N1–Hg1–N2	115.3(2)	N1–Hg1–N2	114.2(4)
N1–Hg1–O2^{i}	96.7(2)	N1–Hg1–Cl1	140.6(1)	N1–Hg1–Br1	137.7(3)
Cl1–Hg1–O2^{i}	114.8(1)	N2–Hg1–Cl1	104.1(1)	N2–Hg1–Br1	108.1(3)
N1–Hg1–O1	72.6(2)	N1–Hg1–O1	71.7(2)	N1–Hg1–O1	71.6(4)
Cl1–Hg1–O1	119.0(1)	N2–Hg1–O1	88.6(2)	N2–Hg1–O1	87.9(4)
O2^{i}–Hg1–O1	82.7(1)	Cl1–Hg1–O1	112.1(1)	Br1–Hg1–O1	111.5(3)
		N1–Hg1–O3	84.3(2)	N1–Hg1–O3	85.1(3)
		N2–Hg1–O3	68.4(2)	N2–Hg1–O3	68.7(4)
		Cl1–Hg1–O3	110.1(1)	Br1–Hg1–O3	110.3(3)
		O1–Hg1–O3	135.7(2)	O1–Hg1–O3	136.7(3)

Symmetry code (i) *x*, –*y*+3/2, *z*+1/2.

Table S2. The hydrogen bond geometry for {[HgCl(pic)]}$_n$ (**1**), [HgCl(pic)(picH)] (**2**) and [HgBr(pic)(picH)] (**3**).

	D–H···A	*d*(D–H)/Å	*d*(H···A)/Å	*d*(D···A)/Å	∠(D–H···A)/°	Symmetry code on A
**2**						
	O4–H41···O2	0.84(1)	1.61(1)	2.451(6)	175(8)	*x*+1, *y*, *z*
**3**						
	O4–H41···O2	0.84	1.61	2.43(1)	163	*x*–1, *y*, *z*

2. IR spectroscopy

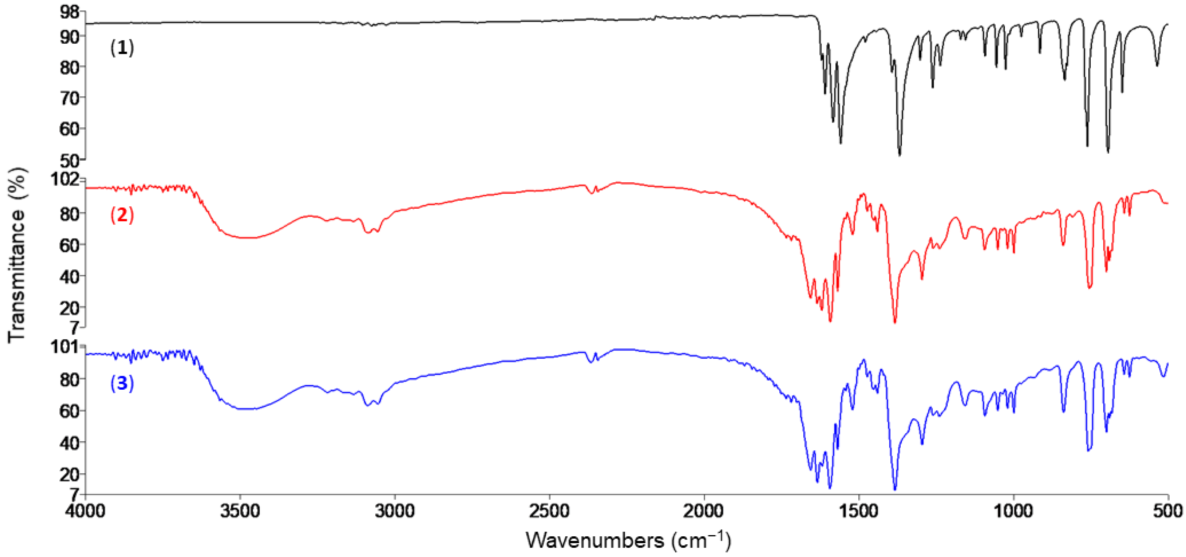

Figure S1. IR spectra of {[HgCl(pic)]}$_n$ (**1**), [HgCl(pic)(picH)] (**2**) and [HgBr(pic)(picH)] (**3**).

3. TG/DSC analysis

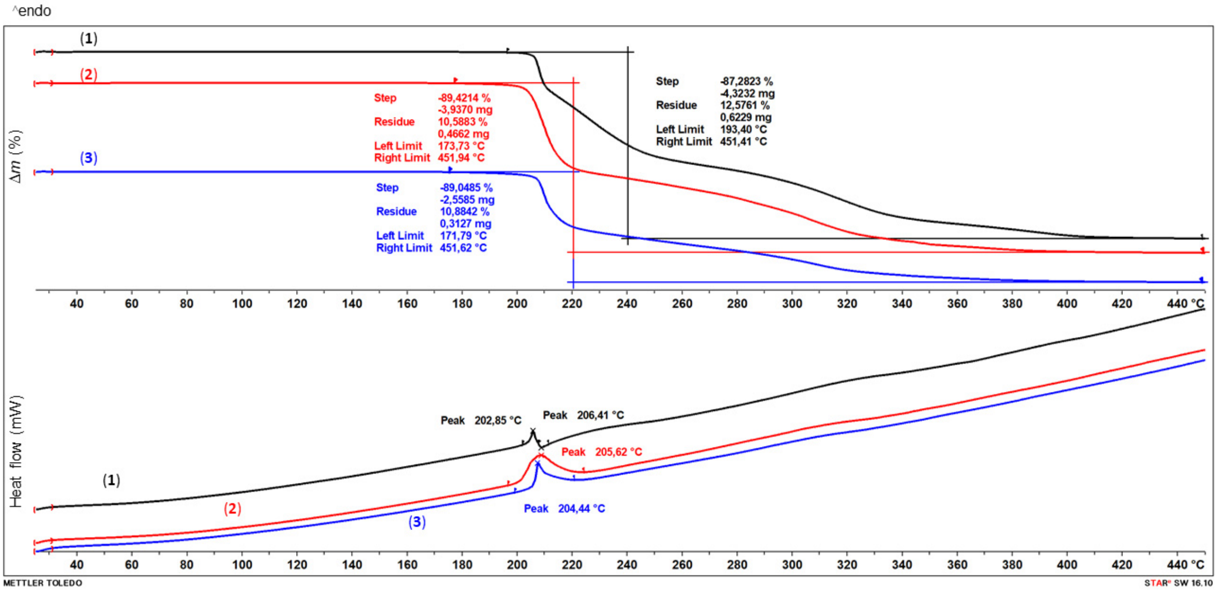

Figure S2. TGA/DSC curves of {[HgCl(pic)]}$_n$ (**1**), [HgCl(pic)(picH)] (**2**) and [HgBr(pic)(picH)] (**3**).
